# Reversing cognitive-decline in older adults in an open-label clinical trial: novel mechanisms and the role of GlyNAC

**DOI:** 10.1093/geroni/igaa057.3157

**Published:** 2020-12-16

**Authors:** Chun Liu, Rajagopal Sekhar, Premranjan Kumar, Charles Minard, Shaji Chacko

**Affiliations:** Baylor College of Medicine, Houston, Texas, United States

## Abstract

Age-associated cognitive-decline is an important risk factor for Alzheimer’s disease, but interventions are lacking. We conducted an open-label trial to test our hypotheses on whether: (1) compared to 8 healthy young adults (25y), 8 ‘healthy’ older adults (74y) have cognitive decline, decreased glucose availability for the brain due to mitochondrial dysfunction, elevated insulin-resistance, oxidative-stress and elevated inflammation; (2) supplementing glycine and N-acetylcysteine (GlyNAC) for 24-weeks corrects deficiency of the endogenous-antioxidant Glutathione and improves these defects, and thereby cognition; (3) stopping GlyNAC supplementation for 12-weeks results in a decline in accrued benefits. Outcome measures included cognitive testing (Montreal cognitive assessment; trail-making tests; verbal-fluency tests; digital-symbol substitution-test), mitochondrial fuel-oxidation, RBC-Glutathione concentrations, plasma oxidative-stress, insulin-resistance and inflammation, and tracer-studies to measure glucose metabolism. Results validated our hypotheses and showed that GlyNAC-supplementation corrected these defects and improved cognition. This trial suggests that supplementing GlyNAC may be important for improving/preventing age-associated cognitive-decline in older adults.

